# Acceptability and perceived utility of different diagnostic tests and sample types for trachoma surveillance in the Bijagos Islands, Guinea Bissau

**DOI:** 10.1093/trstmh/traa179

**Published:** 2021-01-14

**Authors:** Ramandeep Singh Sahota, Salimato Sanha, Anna Last, Eunice Cassama, Adriana Goncalves, Ann H Kelly, Alice Street, Emma M Harding-Esch

**Affiliations:** Faculty of Infectious and Tropical Diseases, London School of Hygiene & Tropical Medicine, London, WC1E 7HT, UK; Programa Nacional de Saúde de Visão, Ministério de Saúde Publica, Bisssau, Guiné Bissau; Faculty of Infectious and Tropical Diseases, London School of Hygiene & Tropical Medicine, London, WC1E 7HT, UK; Programa Nacional de Saúde de Visão, Ministério de Saúde Publica, Bisssau, Guiné Bissau; Faculty of Infectious and Tropical Diseases, London School of Hygiene & Tropical Medicine, London, WC1E 7HT, UK; Faculty of Social Science and Public Policy, King's College London, London, WC2B 4BG, UK; School of Social and Political Science, Edinburgh University, Edinburgh, EH8 9LD, UK; Faculty of Infectious and Tropical Diseases, London School of Hygiene & Tropical Medicine, London, WC1E 7HT, UK

**Keywords:** *Chlamydia trachomatis*, community engagement, diagnostic, Guinea Bissau, qualitative, trachoma

## Abstract

**Background:**

Trachoma is the leading infectious cause of blindness worldwide and is nearing elimination as a public health problem in Guinea Bissau. It is imperative that elimination is followed by a successful postvalidation surveillance programme. The aim of this study was to determine the acceptability and perceived utility of different diagnostic tests and sample types that could be used for postvalidation trachoma surveillance in the Bijagos Islands, Guinea Bissau.

**Methods:**

Semistructured interviews with community members and stakeholders involved in trachoma elimination were followed by focus group discussions with community members, covering experiences with trachoma and views on trachoma diagnostic methods and sample types.

**Results:**

In this setting, all diagnostic tests and sample types used for trachoma surveillance were generally considered acceptable by communities. A preference for laboratory-based testing and finger-prick blood samples was expressed as these results were considered more accurate and applicable to a range of diseases beyond trachoma.

**Conclusions:**

Appropriate community and stakeholder engagement and communication regarding the purpose and processes around diagnostic practice prior to trachoma programme implementation are crucial for long-term successful disease-elimination efforts.

## Introduction

Trachoma, one of 20 neglected tropical diseases (NTDs), is the leading infectious cause of blindness worldwide.^1^ It is caused by recurrent infection with ocular strains of the bacterium *Chlamydia trachomatis.* Trachoma initially presents as a follicular conjunctivitis, often accompanied by conjunctival inflammation. Over time, repeat infections can result in fibrosis and scarring of the conjunctiva, inturned eyelashes that scratch the eyeball (trichiasis), corneal opacification and ultimately blindness.^2^ Trachoma has been targeted for global elimination as a public health problem through the WHO-endorsed SAFE strategy (surgery, antibiotics, facial cleanliness and environmental improvement).^3^ For global elimination of trachoma as a public health problem to be reached and maintained, full implementation of the SAFE strategy is required.^4^

WHO recommends clinical examination of the upper tarsal conjunctiva to diagnose active trachoma, using the WHO-simplified grading system, which provides an easy and reliable method to classify the disease at the community level.^5^ The prevalence of the clinical signs of trachomatous inflammation, follicular (TF) and trachomatous trichiasis (TT), determine whether trachoma is a public health problem, defined as prevalences at the evaluation unit level of ≥5% TF in children aged 1–9 y and/or ≥0.2% TT unknown to the health system in adults aged ≥15 y. The prevalence of TF also determines the number of rounds of annual mass drug administration (MDA) with antibiotics that are required before prevalence is re-evaluated.^6^

However, there is poor correlation between the clinical signs of TF and evidence of *C. trachomatis* infection, with the prevalence of TF higher than that of infection, especially post-MDA.^7^ Consequently, alternative markers for ocular *C. trachomatis* detection have been developed and evaluated, including laboratory-based assays and point-of-care tests (POCTs),^8–11^ as these may be more appropriate and sustainable indicators for trachoma surveillance, especially after elimination validation. These methods require the collection of samples: conjunctival swabs for antigen or nucleic acid-based detection tests, or finger-prick blood samples for serology.^10^

The aim of this study was to determine the acceptability and perceived utility of different diagnostic tests and sample types that may have future application in trachoma surveillance in the Bijagos Islands, Guinea Bissau. The Bijagos Islands are a remote archipelago of islands lying off the coast of Guinea Bissau. Trachoma has been shown to be a public health problem on the archipelago, with the national baseline trachoma survey conducted in 2005 estimating TF prevalence in 1–9 y-olds to be 21.4% and TT prevalence in ≥15 y-olds to be 7.7%.^12^ Subsequent cross-sectional population-based prevalence surveys found the prevalence of TF and *C. trachomatis* in 1–9 y-olds and TT in ≥15 y-olds to be 22.0%, 25.4% and 3.5%, respectively, in 2012,^13^ and 7.4%, 6.6% and 5.1%, respectively, in 2014 after a single round of MDA.^14^ These prevalences exceed WHO thresholds for trachoma elimination. However, following several years of SAFE implementation during 2012–2016, including MDA with azithromycin for active trachoma and ∼800 TT surgeries in total since 2015, the Bijagos Islands are now nearing the elimination thresholds.^15^ The Bijagos Islands have also been the location for research involving the collection of conjunctival samples in addition to clinical examination for trachoma diagnosis^13,16,17^ and blood samples for the detection of sexually transmitted infections (STIs).^18^ They were therefore an ideal setting for this study.

## Materials and Methods

### Study design

Semi-structured interviews (SSIs) were held with two groups, community members in Bubaque, the island in the Bijagos archipelago with the largest population, and key stakeholders working on the Guinea Bissau trachoma programme. The SSIs were followed by focus group discussions (FGDs) with community members in Bubaque. All data were collected in June and July 2018.

### Recruitment of participants

Community SSI participants were sampled using a purposive snowball technique, whereby in each community, the community leader was identified and invited to participate in the study, and was then asked to identify others to participate in the study. Additional community residents were also approached for interview to assess the diversity of views within the community. We aimed to recruit equal numbers of male and female participants across a wide range of ages, with recruitment ending when theme saturation was reached, suggesting that no new themes would be identified with the inclusion of additional participants.^19^ For stakeholder SSIs, a stakeholder mapping exercise was conducted to identify individuals who were involved in the national and regional trachoma programme.

FGDs took place in the community. FGD participants were recommended and invited to participate by the community leader and were stratified by age and gender to avoid any undue influence to respondents’ participation based on other FGD participant gender and age. Each FGD consisted of three or four people, was held in the community and lasted for ≤1 h.

### Data collection and management

Following written informed consent, SSIs and FGDs were conducted with the support of a predesigned topic guide (Supplementary Information 1 and 2). Different topic guides were used for each interview group as different information was being collected. Questions were open-ended, with additional follow-up questions to further explore interviewees’ reasoning and to encourage information-rich replies. In SSIs and FGDs, community members were asked about previous experiences with trachoma, their relationship with health services and health workers, the acceptability and perceived utility of eye examinations for clinical signs, conjunctival sample collection with a cotton bud and blood sample collection. Stakeholders were additionally asked about how trachoma is diagnosed in the Bijagos Islands and the plan for surveillance of trachoma postelimination. All SSIs and FGDs were recorded with a dictaphone. Community SSIs and FGDs took place in Creole and dictaphone recordings were translated and transcribed verbatim by a local English professor. All transcriptions were checked by a native English speaker to ensure translations were grammatically correct. Stakeholder SSIs took place in English. Same-day translation, transcription and analysis took place to identify constructs of interest and emerging themes, as well as limitations with the data collection process (e.g. certain scientific terms that were unfamiliar to participants), which were evaluated to adapt future SSIs and FGDs as necessary.

Community SSIs and FGDs continued until it was deemed that theme saturation had been reached. As there was a finite number of stakeholders involved in the trachoma programme, no further stakeholder interviews took place once these individuals had been interviewed.

### Framework analysis

To gain insight into the salient themes emerging from the qualitative data, a framework approach was employed.^20^ Combining grounded theory with an a priori thematic matrix, framework analysis allows for the iterative development and elaboration of themes that correspond to specific questions.^21^ Firstly, transcriptions were compared, and common themes and subthemes were established by reading and rereading through transcriptions and highlighting passages of text. Within those frequently occurring, similarities and differences were identified, with coding schemes developed to label the themes and allow any other subthemes or trends in the data to be detected and indexed. The emergent themes were refined through an iterative process of data familiarisation, coding and indexing into a predefined set of categories, including disease awareness, examination experiences, acceptability of different sample types and perceived programme utility; this allowed for a summary of themes to emerge from the qualitative data.^21,22^ Emerging themes identified through analysis were examination anxiety, diagnostic credibility and hierarchies of clinical trust.

## Results

### Summary of participants

Forty-two community SSIs were conducted across nine communities with an equal number (n=21) of male and female participants. The age range of SSI participants was 19–95 y with more 50–69-y-old females and more 70–99-year-old males (Table 1).

**Table 1.** Summary of community member semistructured interview numbers by community, gender and age range

	18–49 y		50–69 y		70–99 y	
Community	Male	Female	Male	Female	Male	Female
1	1	-	2	2	-	-
2	-	-	3	-	-	-
3	-	1	1	2	-	-
4	-	-	-	3	1	-
5	2	-	2	-	-	-
6	-	-	1	-	-	-
7	-	3	-	1	-	-
8	2	1	-	6	4	1
9	2	1	-	-	-	-
Total	7	6	9	14	5	1

Six FGDs were conducted across three communities. In each community, one FGD was formed entirely of male participants and the other entirely of female participants. The age range of participants was 18–45 y (Table 2).

**Table 2.** Summary of community member focus group discussion numbers by community, gender and age range

	18–29 y		30–39 y		40–45 y	
Community	Male	Female	Male	Female	Male	Female
1	-	1	3	-	-	2
2	3	3	1	-	-	-
3	1	3	1	1	1	-
Total	4	7	5	1	1	2

Five stakeholder SSIs were conducted. The breakdown of interviewees by professional role was one ophthalmic doctor, one ophthalmic nurse, one public health doctor and two non-governmental organisation (NGO) representatives.

### Eye examination and clinical diagnosis

Twenty-six (62%) community SSI respondents had experienced an eye examination for trachoma diagnosis. Nine of these (35%) had negative feedback on the use of a light to check for clinical signs, specifically its brightness and the pain this caused both during and after the examination.

*The light they used was strong to my eyes. After that I took maybe one hour without seeing well* (female community member, 59 y, SSI).

Twelve (29%) community SSI participants doubted the utility of an eye examination to diagnose trachoma. They were unsure whether simply looking at someone's eyes was sufficient, suggesting that they would feel more confident about their diagnosis if more modern methods had been utilised.

*For me it is not good. There is no machine. I prefer a machine because it is possible to see which problems somebody has* (female community member, 60 y, SSI).

Stakeholders were supportive of clinical diagnosis as the primary method of diagnosis, reporting that it was quick, easy and useful.

*It is a test that is in line with WHO guidelines. It is in line with best practice. It is happening all around the world and of course in trachoma-endemic countries. It is very feasible, anyone can be trained to do it, it is not too difficult to do* (NGO representative).

*This is the best way that we have to check, especially when we are doing fieldwork* (ophthalmic nurse).

The drawbacks of eye examinations elucidated by stakeholders include their subjectivity, the standardised training needed before graders can conduct fieldwork and the neglect that is shown by graders towards personal hygiene.

*It can be subjective…If there is poor personal hygiene it can introduce infection instead of helping the patient* (NGO representative).

### Sample collection

#### Upper conjunctival swab

Most community members were in favour of conjunctival samples, however, six (14%) community SSI participants expressed concerns surrounding their safety and the potential pain and damage that may be caused by a cotton bud's close proximity to the surface of the eye.

*The way they open the eyes makes us afraid. If they use that stick they will damage the eyes* (female community member, 18 y, FGD).

Stakeholders suggested that conjunctival samples were feasible to carry out in the field and would be accepted by communities.

*I think it is relatively easy once you are trained. The swabs are available and the patients cooperate. It should be easy to collect the samples* (NGO representative).

#### Blood sample

Three (7%) community SSI participants considered a blood test necessary to diagnose trachoma, preferring it to any other method for trachoma diagnosis. This was supported in FGDs.

*If they don't take out blood, how can they discover what you have?* (female community member, 25 y, FGD).

In community SSIs, there were suggestions that blood samples were preferable to both eye examinations and conjunctival samples because they did not involve health professionals being in close proximity to patients’ eyes.

*Eyes are too sensitive and we are afraid to lose them. I prefer to take out blood instead of touching my eyes* (female community member, 43 y, SSI).

### Laboratory testing

The benefits of laboratory testing to aid diagnosis were highlighted by stakeholders who recognised that the presence of follicles is not always pathognomic for trachoma and that laboratory tests can improve both diagnostic sensitivity and specificity.

*I prefer the lab because if you see somebody's eyes you can't say that it is trachoma, you need to test it in the lab to see if it is or not. Three or four follicles doesn't mean that it is not trachoma. Sometimes there are no symptoms* (ophthalmic doctor).

Community members identified that blood or conjunctival swab samples allowed the use of the laboratory in trachoma diagnosis, which was perceived to provide more credible results.

*For me the one they will take to the lab is good because it is impossible to use that light to find any disease. I think the lab is better because they will use a machine to investigate the disease* (male community member, 19 y, FGD).

Stakeholders also thought that taking samples with a cotton bud enabled laboratory testing to be conducted and was a useful, feasible procedure.

*I think it is helpful if you take it to the laboratory. When you take the sample to the laboratory it confirms the bacteria. All of these tests are complementing each other* (public health doctor).

Eight (19%) community SSI participants thought that a blood test would be advantageous because the sample can be used to detect other diseases in addition to trachoma.

*This one is the best one because maybe they can discover another disease. I think they can discover a lot of diseases including malaria* (male community member, 19 y, SSI).

Most community participants supported the idea of samples being sent to the laboratory for testing in favour of a same-day diagnosis. They believed that it was important to give the doctor time and space to make a diagnosis, suggesting they would place greater faith in the diagnosis if they knew it had taken some time to come to the result.

*You can't ask someone and he gives you answer in the same day. He has to sit and think about the answer* (male community member, 42 y, SSI).

While stakeholders were optimistic about the progress that could be made with laboratory testing, they were also cautious about the feasibility of its implementation, particularly from a resource standpoint.

*There may be some health system strengthening needed…There will be additional resources needed to provide the equipment, to build capacity, to develop skills of staff. It is not easy, but is worthwhile. It's a good investment if it gives better results. We need to invest in the health system* (NGO representative).

### Doctors vs nurses

Twenty-six (62%) community SSI participants indicated a preference for having a doctor carry out a diagnosis or sample collection.

*I prefer a doctor; it doesn't mean that the nurses are not good but the doctors have more knowledge about what they are doing* (male community leader, 58 y, SSI).

### Local doctors vs foreign doctors

Half of community SSI respondents (n=21) insinuated that communities had a predilection to being seen by a foreign doctor compared with a local doctor, with foreign doctors being associated with greater experience and knowledge.

*Local doctors tried to help [but] didn't succeed, so now I prefer a foreign doctor to do it* (male community member, 29 y).

Some people did not like the idea of being seen by a foreign doctor who stays for a short period of time before returning to their home country. These participants placed a greater emphasis on the continuity of care, which they believed could only be provided by a local doctor.

*We think that the local doctor is better because he will stay here and take care of us. The foreign doctor will come and then leave* (male community member, 19 y).

## Discussion

This study of community and stakeholder opinions of trachoma diagnosis demonstrated that although clinical eye examination was acceptable to most, there was a preference for sample types that did not involve close proximity with the eye and that enabled laboratory diagnosis. Furthermore, greater confidence in doctors vs nurses, and foreign vs local doctors, was expressed, although appreciation of the continuity of care provided by local doctors was acknowledged.

There were several limitations to this study. First, community participants were recommended by the community leader, which may have led to biased results if only participants in favour with the community leader were interviewed. Second, a field translator was needed for community SSIs and FGDs, and later for transcript translation. This likely impacted on the ability to ask appropriate follow-up questions during the fieldwork discussions, and nuances in responses may have been lost in translation. Third, although all of the stakeholders based in Guinea Bissau were reachable, international stakeholders were not contactable during the data collection period of this study. As such, the results presented lack a global perspective. Local geographical representativeness is also limited, as only community participants from Bubaque were recruited; different responses may have been obtained from other Bijagos Islands, particularly from those with less trachoma research study familiarity. There are limited data comparing the prevalence of trachoma on Bubaque relative to the other Bijagos Islands, and of the Bijagos Islands to mainland Guinea Bissau; although research studies have been based in up to seven islands, reported prevalence results are aggregated.^13,14,16,17^ The Bijagos Islands are generally homogeneous sociodemographically, with >90% belonging to the Bijogo ethnic group.^13^ However, there are differences in amenities and access between Bubaque, which is the main port access between mainland Guinea Bissau and the Bijagos Islands, and the other islands, such that it would be valuable to extend this work to other islands in the archipelago. The rural lifestyle is similar to other coastal areas in West Africa, but the Bijogo ethnic group is culturally distinct from others on the mainland, perhaps reducing the generalisability of these results.^23^ Fourth, only adults were recruited, whereas diagnosis of active trachoma is targeted towards children (specifically 1–9 y-olds, as per WHO recommendations).^3^ Children's preferences of clinical examination vs sample collection for laboratory diagnosis may differ from those of the adult respondents in this study.

This study also had a number of strengths. The views of community members as well as key stakeholders involved in the trachoma elimination programme were explored. Furthermore, community member perspectives were explored via both SSIs and FGDs, enabling triangulation of results. Saturation was reached early (FGDs were not warranted in the last six communities), showing agreement in results, and that results were representative of the communities the study sample represents.

In general, clinical examination was acceptable to both community members and stakeholders. Clinical examination is the current WHO-recommended diagnostic method, with a rigorous standardisation process in place to certify grader competence in population-based prevalence surveys and the development of new tools to further aid diagnostic accuracy.^24,25^ However, it is also well recognised that clinical signs are poorly correlated with ocular *C. trachomatis* infection, especially after MDA.^7^ Furthermore, as countries move towards postvalidation surveillance, more sustainable diagnostic methods that can be integrated into existing national health systems are needed, especially as it becomes increasingly difficult to train and certify trachoma graders as trachoma prevalence decreases.^25^ Ocular or blood samples that enable laboratory detection are primary ‘alternative indicator’ candidates,^10^ as is the use of photography.^26^

The fear around eye swabs points to the critical importance of trusting the practitioner performing the procedure, which reflects cultural and social norms (e.g. preference for doctors over nurses). These norms within which interventions are implemented must be understood so that programmes can be optimally successful.^27^ The individual who is communicating with the community, and the quality of the information they provide, are important.^28^ Our results suggest that in the Bijagos Islands, individuals higher up in the ‘hierarchy’ (well-respected doctors) would be best placed to do this. There is evidence from the UK to suggest that when a patient does not see their preferred healthcare professional, reduced levels of trust, confidence and communication are felt by the patient.^29^ However, given that there is only one ophthalmologist responsible for the Bijagos Islands, it is not feasible for them to always conduct trachoma diagnosis. Furthermore, preference for foreign over local doctors is not sustainable, nor is it to the healthcare system's benefit to be reliant on incoming personnel, rather than investing in strengthening in-country capacity. Stakeholders and community members should instead develop a pathway for ongoing feedback, allowing for the development of a rapport which will aid a relationship of mutual trust.

The preference for sample collection enabling laboratory diagnosis was principally due to greater confidence in the objective results produced by machines and the ability to test for multiple infections. Finger-prick blood samples were preferred over eye swabs, possibly due to familiarity with blood sample collection for malaria diagnosis, which is highly endemic in Guinea Bissau.^30^ As a result, large-scale implementation of blood sample collection should be acceptable and feasible, as the materials and skilled staff are already in place.

Along with a preference for modern laboratory-based diagnostic techniques, there was the need for time to make an accurate diagnosis. Thus, more emphasis was placed on test accuracy than speed of result. This resonates with research conducted in the field of STI POCTs, where patients’ willingness to wait for results depended on their reason for clinic attendance.^31^ However, limitations to the routine implementation of these novel diagnostic methods depends on evidence to support their utility and the capacity to implement them as routine. The main barriers highlighted to carrying this out at scale are the need for an improved infrastructure, training of fieldworkers and having the facilities and resources to carry out testing. If these alternative indicators to clinical diagnosis become the internationally agreed methods for postvalidation surveillance, mechanisms for implementing them at little or no additional cost to health ministries will need to be identified.^11^ This is in line with the 2021–2030 NTDs roadmap, which seeks to meet the 2030 targets in part through integration and mainstreaming cross-cutting approaches into national health systems, increasing cost-effectiveness and programme coverage.^32^

### Conclusions

Community members and stakeholders were largely accepting of all trachoma diagnostic methods. However, community members expressed fears relating to practices directly involving the eye, resulting in a preference for blood sample collection. All respondents perceived benefits of laboratory testing over clinical examination. For these alternative indicators for trachoma diagnosis to become feasible at scale, integration into national health systems is needed, along with investment in resources, personnel and infrastructure.

## Supplementary Material

traa179_Supplemental_Files

## Data Availability

The data underlying this article will be shared on reasonable request to the corresponding author.
