# Supplementary material for: Acceptability and perceived utility of different diagnostic tests and sample types for trachoma surveillance in the Bijagos Islands, Guinea Bissau
Source: Trans R Soc Trop Med Hyg. 2021 Jan 14;115(8):847–53. doi: 10.1093/trstmh/traa179 (PMC8382514; doi:10.1093/trstmh/traa179)
Supplement: traa179_Supplemental_Files [file traa179_Supplemental_Files.zip › Supplementary Information 2 FGD Topic Guide.docx]

## Focus Group Discussion Topic Guide

*Acceptability and utility of different diagnostic tests and sample types for trachoma surveillance in the Bijagos Islands, Guinea Bissau*

Groups to last 30 to 45 minutes.

Introduction explaining what trachoma is and how it is tested for. Use local language to describe what trachoma is as it can be confused with other eye diseases.

Have you ever been tested for trachoma? Can you tell me about this?

Follow-Up: When? Where? What was done and by whom? What did you think about this experience? Would you do it again? Did it affect your daily routine? Was there anything in particular that you didn’t like or made you feel uncomfortable? What time of year would be the best time for you? Do you know anyone else who has been tested for trachoma?

Introduction to different sample types used in trachoma.

What do you think about having your eyes examined to check for the presence of trachoma?

Follow-Up: Why? In what setting or surrounding would they feel comfortable having this examination carried out? Who would you feel most comfortable carrying out an examination of this kind?

Prompt: Image of someone having their eyes examined for trachoma.

What do you think about having your eyelid swabbed to check for the presence of trachoma?

Follow-Up: Why? In what setting or surrounding would they feel comfortable having this examination carried out? Who would you feel most comfortable carrying out an examination of this kind?

Prompt: Image of someone having their eyes swabbed for trachoma.

What do you think about having a blood sample taken to check for the presence of trachoma?

Follow-Up: Why? In what setting or surrounding would they feel comfortable having this examination carried out? Who would you feel most comfortable carrying out a procedure of this kind?

Prompt: Image of someone having their blood taken for trachoma.

Order the following cards in terms of how acceptable they are to you.

Large cards with the following pictures: eye examination, eyelid swab, blood sample

Any prompts for discussion.

Introduction to different methods of diagnosis used in trachoma.

What are some important issues for you regarding the diagnosis of trachoma?

What do you think about being diagnosed with trachoma by an examination of your eye?

Follow-Up: Why? Is there anything about this that makes you feel uncomfortable? Is there anything about this that you would change?

What do you think of being diagnosed with trachoma by a point of care test?

Follow-Up: Why? Is there anything about this that makes you feel uncomfortable? Is there anything about this that you would change?

What do you think about being diagnosed with trachoma by a sample that is taken to a laboratory?

Follow-Up: Why? Is there anything about this that makes you feel uncomfortable? Is there anything about this that you would change?

Would you rather be diagnosed by examination or after a sample has been taken from you?

Follow-Up: Why?

Would you rather receive your diagnosis straight away or after testing has taken place in a laboratory?

Follow-Up: Why?

Close session with any final concerns from the group.
