# Supplementary material for: Acceptability and perceived utility of different diagnostic tests and sample types for trachoma surveillance in the Bijagos Islands, Guinea Bissau
Source: Trans R Soc Trop Med Hyg. 2021 Jan 14;115(8):847–53. doi: 10.1093/trstmh/traa179 (PMC8382514; doi:10.1093/trstmh/traa179)
Supplement: traa179_Supplemental_Files [file traa179_Supplemental_Files.zip › Supplementary Information 1 SSI Topic Guides.docx]

## Community Semi-Structured Interview Topic Guide

## *Acceptability and utility of different diagnostic tests and sample types for trachoma surveillance in the Bijagos Islands, Guinea Bissau*

Good morning, my name is Raman. Thank you for agreeing to speak with me today.

We are here today to talk about trachoma, a disease that affects the eye. I will be asking you a few questions about how this disease is tested for. I want to get your opinions on this. There is no right, wrong or undesirable answer. I would like you to feel comfortable saying what you really think and how you really feel.

Just to remind you, I will be tape recording this conversation so that I do not miss anything that you have said.

Have you ever been tested for trachoma? Can you tell me about this?

Follow-Up: When? Where? What was done and by whom? What did you think about this experience? Would you do it again? Was there anything in particular that you didn’t like or made you feel uncomfortable?

Do you know anybody else who has been tested for trachoma? Can you tell me about this?

Follow-Up: When? Where? What was done and by whom? What did you think about this experience? Would you do it again? Was there anything in particular that you didn’t like or made you feel uncomfortable?

How would you describe your other contacts with health services?

Follow-Up: When? Where? What was done and by whom? What did you think about the experience? Did it put you off seeking healthcare? Was there anything in particular that you didn’t like or made you feel uncomfortable?

How would you describe your relationship and interactions with healthcare workers?

Follow-Up: Domestic and International? Male and female?

What do you think about having your eyes examined to check for the presence of trachoma?

Follow-Up: Why? In what setting or surrounding would they feel comfortable having this examination carried out? Who would you feel most comfortable carrying out an examination of this kind?

Prompt: Image of someone having their eyes examined for trachoma.

What do you think about having your eyelid swabbed to check for the presence of trachoma?

Follow-Up: Why? In what setting or surrounding would they feel comfortable having this examination carried out? Who would you feel most comfortable carrying out an examination of this kind?

Prompt: Image of someone having their eyes swabbed for trachoma.

What do you think about having a blood sample taken to check for the presence of trachoma?

Follow-Up: Why? In what setting or surrounding would they feel comfortable having this examination carried out? Who would you feel most comfortable carrying out a procedure of this kind?

Prompt: Image of someone having their blood taken for trachoma.

If you had to choose between an examination, a swab of your eyelid and a blood test which would you prefer?

Follow-Up: Why?

Thank you.

## Stakeholder Semi-Structured Interview Topic Guide

*Acceptability and utility of different diagnostic tests and sample types for trachoma surveillance in the Bijagos Islands, Guinea Bissau*

Good morning, my name is Raman. Thank you for agreeing to speak with me today.

We are here today to talk about the different diagnostic methods and sample types that are used for trachoma surveillance in the Bijagos Islands. The purpose is to get your views on the different diagnostic methods and sample types, and your perceptions on how acceptable and feasible they are. There are no right, wrong or undesirable answers to the questions being asked today. I would like you to feel comfortable saying what you really think and how you really feel. Your perspective on this topic is valuable to us as we try to assess the acceptability and utility of different diagnostic methods and sample types that are used in the surveillance of trachoma.

Just to remind you, I will be tape recording this conversation so that I do not miss anything that you have said.

Which organisation are you associated with and what is your role within this organisation?

Follow-Up: Could you tell me about the role of this organisation in the surveillance of trachoma in the Bijagos Islands? How did you get involved?

Can you tell me about how trachoma is currently diagnosed in the Bijagos Islands?

Follow-Up: What has your organisation’s experience of diagnosing for trachoma been? What are the main challenges for diagnosing trachoma in the Bijagos Islands? Is only one method used or are different methods used for different cases? Why are these methods used?

Can you tell me about which sample types are used for the surveillance of trachoma in the Bijagos Islands?

Follow-Up: What has your organisation’s experience of using these sample types been? Is this the only sample type used? Why is this sample type used?

What is the long term plan for the surveillance of trachoma, i.e. once elimination has been achieved?

Follow-Up: Why? What is the rationale behind this plan? How feasible is it? Do you think this is the best plan or would you suggest something else?

What do you see as the main challenges in the surveillance of trachoma in the Bijagos Islands?

Follow-Up: How can these challenges be overcome? What is the role of the relationship with the community in this?

What do you think about using examination of the eyelid to check for the presence of trachoma?

Follow-Up: Why? Do you and your organisation consider this procedure acceptable? Do you and your organisation consider this procedure useful? Are there any drawbacks to using this procedure?

What do you think about using a swab of the eyelid to check for the presence of trachoma?

Follow-Up: Why? Do you and your organisation consider this procedure acceptable? Do you and your organisation consider this procedure useful? Are there any drawbacks to using this procedure?

What do you think about using a blood sample to check for the presence of trachoma?

Follow-Up: Why? Do you and your organisation consider this procedure acceptable? Do you and your organisation consider this procedure useful? Are there any drawbacks to using this procedure?

If you were having a trachoma diagnosis carried out on you, which sample type would you prefer? Examination of your eyelid, swab of your eyelid or blood sample? Why?

If you were having a trachoma diagnosis carried out on you, which diagnostic method would you prefer? Clinical signs, lab NAAT, rapid test, point of care test or serology? Why?

Do you have any further concerns or comments to make on the different diagnostic tests and sample types used for the surveillance of trachoma in the Bijagos Islands?

Is there anything else you would like to add?

Thank you for your time.
